# *Zavoticus yini* gen. et sp. nov., a New Euaesthetine Rove Beetle From Mid‐Cretaceous Kachin Amber (Coleoptera: Staphylinidae)

**DOI:** 10.1002/ece3.71960

**Published:** 2025-09-07

**Authors:** Yan‐Da Li, Dave J. Clarke, Alfred F. Newton, Di‐Ying Huang, Chen‐Yang Cai

**Affiliations:** ^1^ State Key Laboratory of Palaeobiology and Stratigraphy, Nanjing Institute of Geology and Palaeontology Chinese Academy of Sciences Nanjing China; ^2^ Bristol Palaeobiology Group, School of Earth Sciences University of Bristol Bristol UK; ^3^ Department of Biological Sciences University of Memphis Memphis Tennessee USA; ^4^ Negaunee Integrative Research Center Field Museum of Natural History Chicago Illinois USA

**Keywords:** Euaesthetinae, fossil, Kachin amber, phylogenetic analysis, rove beetle

## Abstract

We describe and illustrate *Zavoticus yini* gen. et sp. nov., a fossil beetle of Euaesthetinae (Staphylinidae) from mid‐Cretaceous Kachin amber, and evaluate its phylogenetic placement based on morphological characters. *Zavoticus* shares several key features with the *Octavius* generic group, including a distinct metendosternal stalk, two pairs of parasclerites on abdominal segments IV–VI, and apically attached abdominal intersegmental membranes. It differs from the two extant genera of the *Octavius* generic group, *Octavius* and *Protopristus*, primarily by having only a very weak and indistinct dorsal nuchal depression. The discovery of *Zavoticus* adds to the limited but growing fossil record of Euaesthetinae and provides new insight into the early diversification of this group.

## Graphical abstract

We describe and illustrate a new euaesthetine rove beetle from mid‐Cretaceous Kachin amber, *Zavoticus yini* gen. et sp. nov. Based on morphological comparisons and a phylogenetic analysis, *Zavoticus* is placed in the *Octavius* generic group.

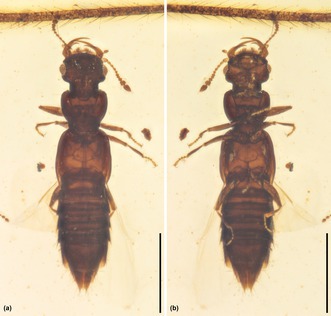

## 1 Introduction

Euaesthetinae is a likely monophyletic subfamily in the rove beetle family Staphylinidae, comprising about 1155 described extant species in 23 genera (Clarke 2018; Newton 2022). This subfamily is most notably characterized by the dentate anterior edge of the labrum, although this feature appears to have been secondarily lost in some members. While numerous euaesthetine species have been described, especially by Volker Puthz (e.g., Puthz 2008a, 2013, 2014a, 2014b), the detailed morphology and the higher‐level relationships among these species have been largely unavailable.

A sister relationship between Euaesthetinae and Steninae has been confirmed by both morphological and molecular studies (Leschen and Newton 2003; Clarke and Grebennikov 2009; McKenna et al. 2015; Lü et al. 2020), but relationships within Euaesthetinae are far less clear. In the current classification scheme (e.g., Herman 2001; Clarke 2018), six tribes are recognized in Euaesthetinae, namely Alzadaesthetini, Austroesthetini, Euaesthetini, Nordenskioldiini, Stenaesthetini, and Stictocraniini (=Fenderiini). However, a morphology‐based phylogenetic study by Clarke and Grebennikov (2009) revealed that Alzadaesthetini, Austroesthetini, Euaesthetini, and Stictocraniini are non‐monophyletic, leaving only Stenaesthetini as potentially monophyletic (Nordenskioldiini was not sampled). Molecular phylogenetic studies have included only a few euaesthetine genera to date, but they corroborate the heterogeneity within Euaesthetini (McKenna et al. 2015; Lü et al. 2020).

The fossil record of Euaesthetinae has been comprehensively reviewed by Clarke (2018). Particularly, regarding the Cretaceous record, Lefebvre et al. (2005) described a species from Lower Cretaceous Lebanese amber, which was originally assigned to a genus of its own, *Libanoeuaesthetus* Lefebvre et al., and subsequently transferred to the extant genus *Nordenskioldia* Sahlberg by Puthz (2008b). Clarke and Chatzimanolis (2009) described a species from mid‐Cretaceous Kachin amber, which was assigned to the extant genus *Octavius* Fauvel. Clarke (2018) also mentioned another undescribed euaesthetine fossil from Kachin amber with affinity to *Octavius*.

In the present study, we describe a new genus and species of Euaesthetinae from mid‐Cretaceous Kachin amber and evaluate its position through phylogenetic analyses.

## 2 Material and Methods

### 2.1 Material

**FIGURE 1:**
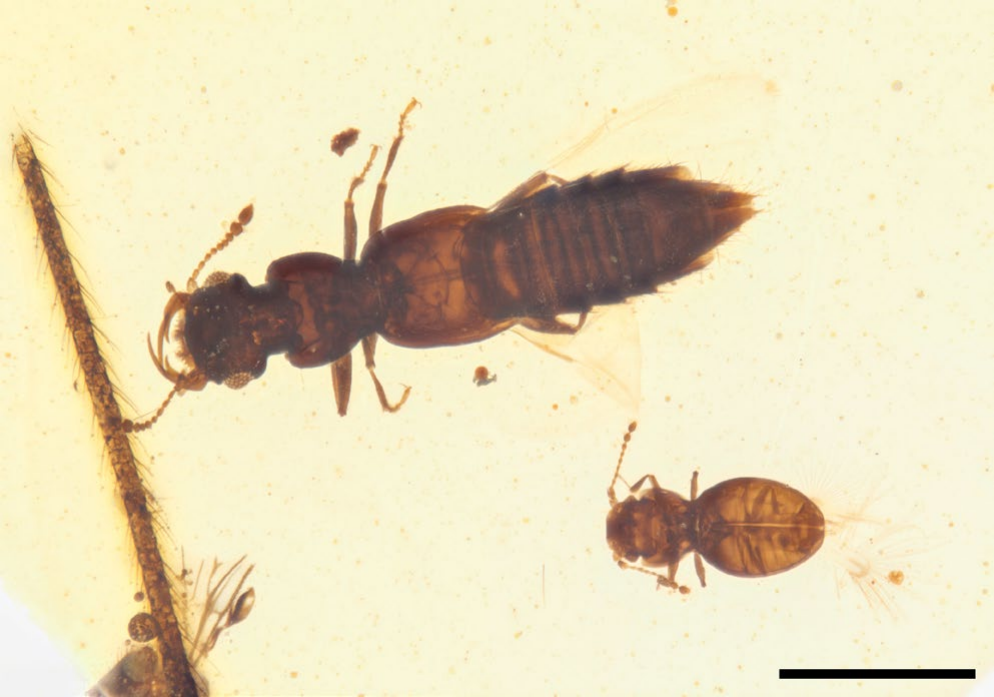
Kachin amber specimen NIGP200739, containing a syninclusion of *Zavoticus yini* sp. nov. and *Kekveus brevisulcatus*. Scale bar: 500 μm.

The Kachin (Burmese) amber specimen studied herein (Figure 1) originated from amber mines near Noije Bum (26°20′ N, 96°36′ E), Hukawng Valley, Kachin State, northern Myanmar. The specimen is deposited in the Nanjing Institute of Geology and Paleontology (NIGP), Chinese Academy of Sciences, Nanjing, China. The holotype of *Zavoticus yini* gen. et sp. nov. (NIGP200739‐2) is preserved along with the previously published holotype of *Kekveus brevisulcatus* Li et al. (NIGP200739‐1) in the same amber piece (Li, Yamamoto, et al. 2023). D.J.C. re‐examined the holotype of *Octavius electrospinosus* Clarke & Chatzimanolis, which has since been embedded in resin by someone at the American Museum of Natural History (AMNH).

### 2.2 Fossil Imaging

Brightfield images were taken with a Zeiss Discovery V20 stereo microscope. Confocal images were obtained with a Zeiss LSM710 confocal laser scanning microscope, using the 488 nm (Argon) or 561 nm (DPSS 561–10) laser excitation line (Fu et al. 2021). The brightfield images were stacked with Helicon Focus 7.0.2 and Zerene Stacker 1.04. The confocal images were manually stacked in Adobe Photoshop CC. Images were further processed in Adobe Photoshop CC to adjust brightness and contrast.

### 2.3 Morphological Terminology

The general morphological terminology follows Lawrence and Ślipiński (2013). The terminology for more specific structures of euaesthetines follows Clarke and Grebennikov (2009) and Clarke (2011).

### 2.4 Phylogenetic Analyses

To evaluate the systematic placement of the new species, constrained analyses were performed under both Bayesian inference (e.g., Li et al. 2022; Li, Ślipiński, et al. 2023) and weighted parsimony (e.g., Li et al. 2024, 2025). The data matrix (File S1) was mainly derived from the previously published dataset by Clarke and Grebennikov (2009), and the character list can be found in Clarke and Grebennikov (2009). The constraining backbone tree was based on Lü et al. (2020), which mainly fixed the relationships among the outgroups, as the internal relationships of Euaesthetinae have not been intensively studied based on molecular data.

The Bayesian inference was performed using MrBayes 3.2.6 (Ronquist et al. 2012). Two MCMC analyses were run simultaneously, each with one cold chain and three heated chains. Trees were sampled every 1000 generations. Analyses were stopped when the average standard deviation of split frequencies remained below 0.01. The first 25% of sampled trees were discarded as burn‐in, and the remainder were used to build a majority‐rule consensus tree. The tree was drawn with the online tool iTOL 6.5.2 (Letunic and Bork 2024) and graphically edited with Adobe Illustrator CC 2017.

The parsimony analysis was performed under implied weights in R 4.1.0 (R Core Team 2021), using the R script provided by Li et al. (2024) (File S2), which deploys the R package TreeSearch 1.6.0 (Smith 2023). The concavity constant was set to 12, following the suggestion by Goloboff et al. (2018) and Smith (2019). Character states were mapped onto the tree with WinClada 1.00.08.

## 3 Systematic Paleontology

Order Coleoptera Linnaeus, 1758.

Superfamily Staphylinoidea Latreille, 1802.

Family Staphylinidae Latreille, 1802.

Subfamily Euaesthetinae Thomson, 1859.

### 3.1 Genus *Zavoticus* gen. nov.

**FIGURE 2:**
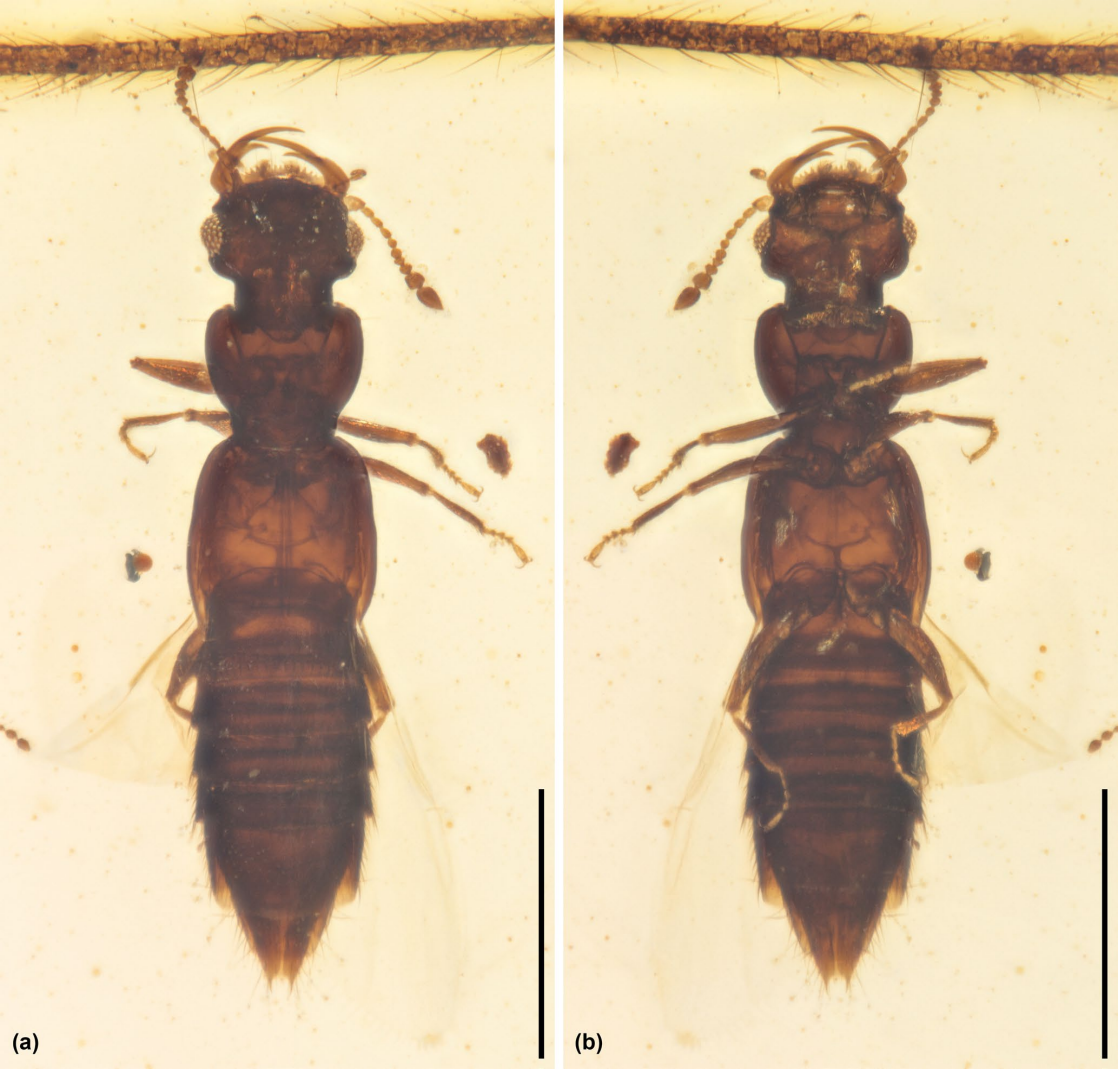
General habitus of *Zavoticus yini* sp. nov., holotype, NIGP200739‐2, under brightfield microscopy. (a) Dorsal view. (b) Ventral view. Scale bars: 500 μm.

**FIGURE 3:**
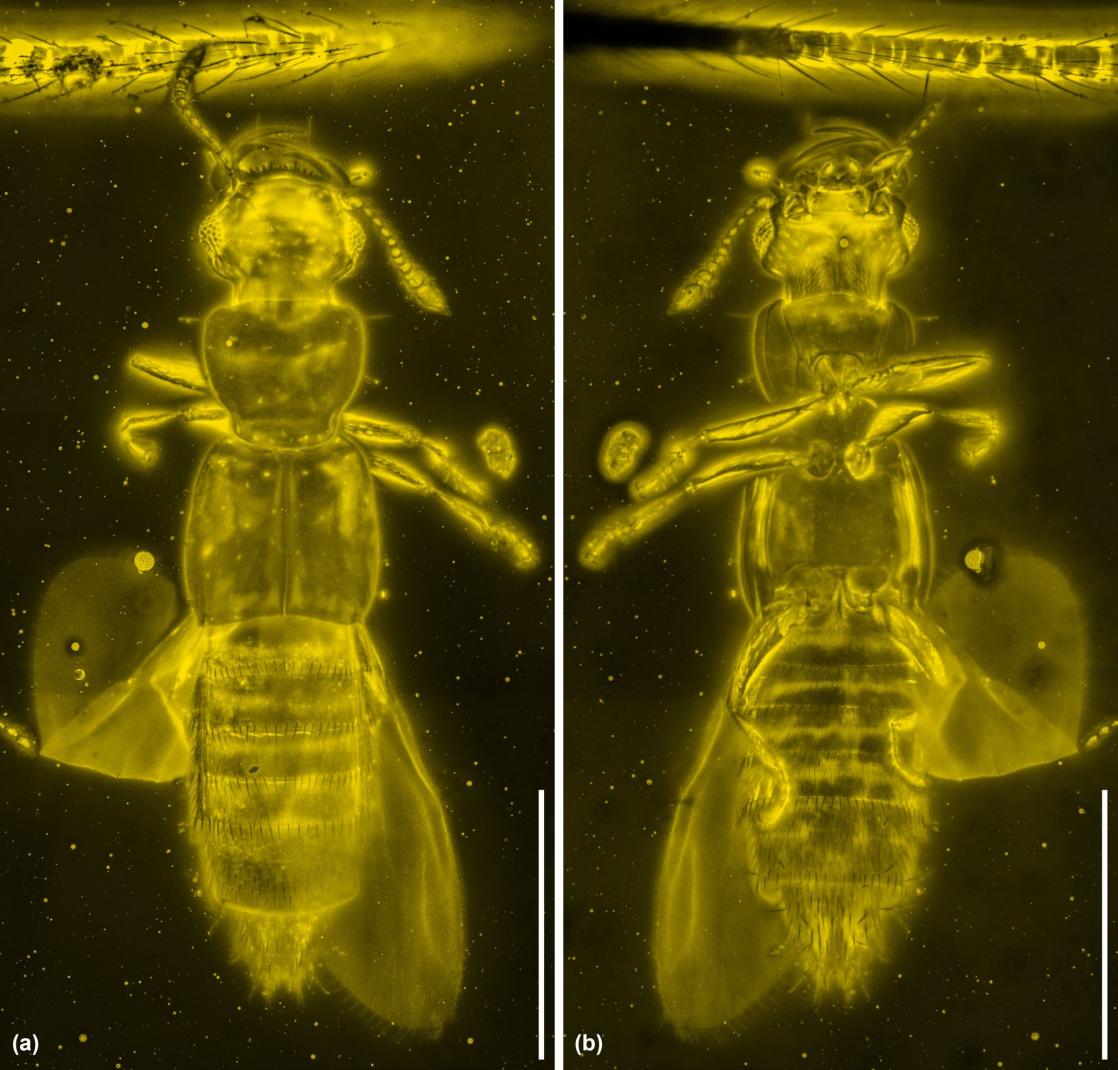
General habitus of *Zavoticus yini* sp. nov., holotype, NIGP200739‐2, under confocal microscopy. (a) Dorsal view. (b) Ventral view. Scale bars: 500 μm.

**FIGURE 4:**
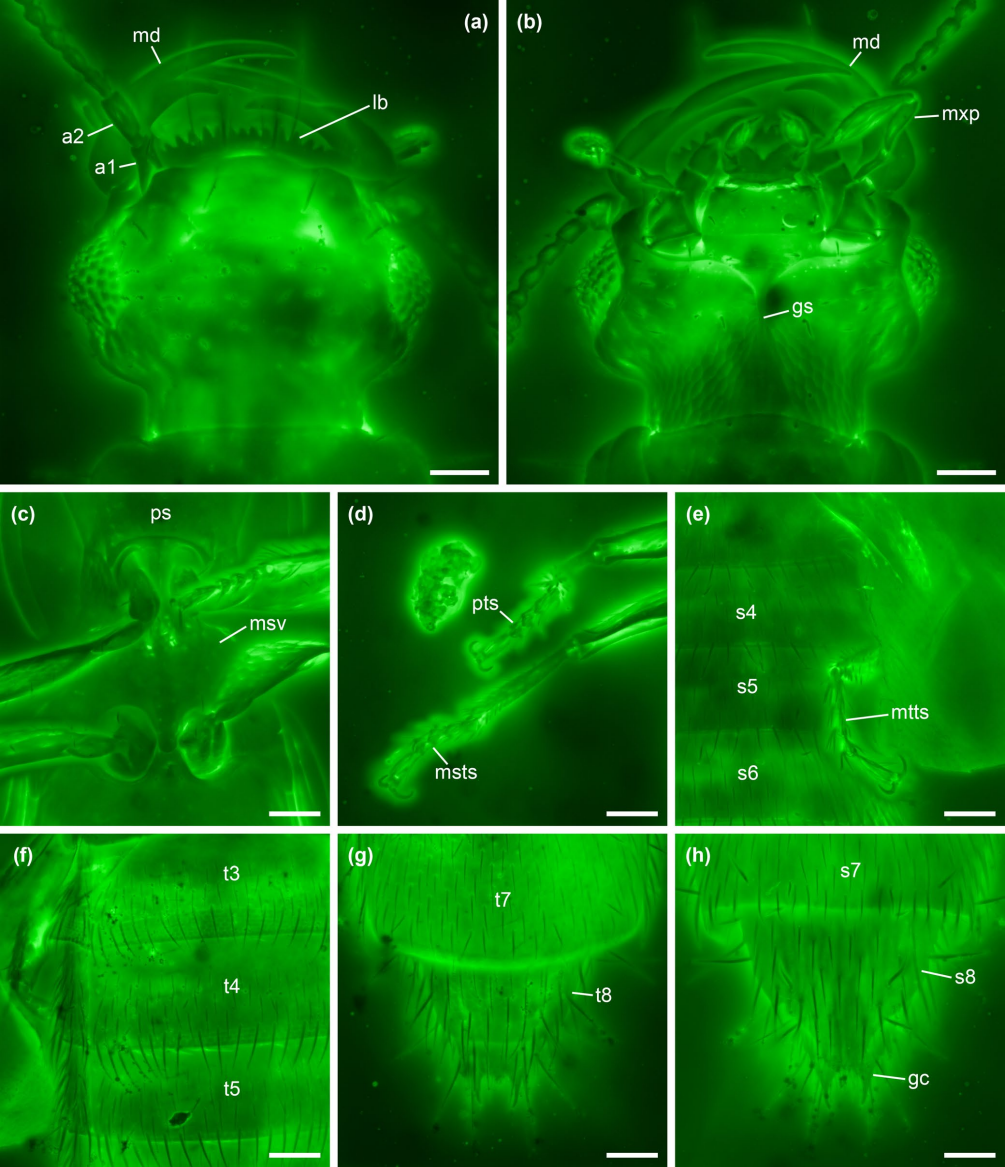
Details of *Zavoticus yini* sp. nov., holotype, NIGP200739‐2, under confocal microscopy. (a) Head, dorsal view. (b) Head, ventral view. (c) Pro‐ and mesothorax, ventral view. (d) Fore and mid legs. (e) Abdomen, ventral view. (f) Abdomen, dorsal view. (g) Abdominal apex, dorsal view. (h) Abdominal apex, ventral view. Abbreviations: a1–2, antennomeres 1–2; gc, gonocoxite; gs, gular suture; lb, labrum; md, mandible; msts, mesotarsus; msv, mesoventrite; mtts, metatarsus; mxp, maxillary palp; ps, prosternum; pts, protarsus; s4–8, abdominal sternites IV–VIII; t3–8, abdominal tergites III–VIII. Scale bars: 50 μm.

**Type species**. *Zavoticus yini* sp. nov. (Figures 2, 3, 4).

**Etymology**. The generic name is an arbitrary combination of letters, inspired by the name *Octavius*. The name is masculine in gender.

**Diagnosis**. Dorsal surface of head, pronotum, and elytra relatively smooth, without distinct punctation or other modifications (Figure 3a). Head dorsally without distinct nuchal groove (Figure 4a). Eyes well developed (Figure 4a). Antennae 11‐segmented, with 2‐segmented club; antennomeres 10 and 11 clearly separated, articulated (Figure 2). Labrum anteriorly coarsely denticulate (Figure 4a). Submentum without transverse carina (Figure 4b). Gular sutures largely united (Figure 4b). Lateral pronotal carina present, complete; hypomeral marginal carina present (Figure 3b). Mesoventrite without oblique carinae and lateral transverse carinae (Figure 4c). Metendosternite with clearly developed stem (Figure 2b). Hind wings well developed (Figure 3). Tarsi 4‐4‐4, simple (Figure 4d,e). Abdomen with two pairs of subparallel‐sided parasclerites at least on segments IV–VI (Figure 4f). Abdominal intersegmental membranes with “brick‐wall” pattern of microsclerites, and attaching apically to preceding segment (Figure 4f). Abdominal sternite III with sharp intercoxal carina (Figure 3b).

### 3.2 *Zavoticus yini* sp. nov.

**Material**. Holotype, NIGP200739‐2, female.

**Etymology**. The species is named after the coleopterist Dr. Zi‐Wei Yin (Shanghai Normal University, China), in recognition of his contributions to the systematics of Staphylinidae.

**Locality and horizon**. Amber mine located near Noije Bum Village, Tanai Township, Myitkyina District, Kachin State, Myanmar; unnamed horizon, mid‐Cretaceous, Upper Albian to Lower Cenomanian.

**Diagnosis**. As for the genus.

**Description**. Body elongate, slender, and more or less parallel‐sided, about 1.5 mm long, 0.35 mm wide. Dorsal surface of head, prothorax, and elytra subglabrous.

Head with distinct lateral neck constriction, dorsally with very weak and indistinct nuchal depression only, without distinct nuchal groove, with temples behind eyes, with two pairs of macrosetae on vertex and frons. Eye moderately large, evenly rounded laterally, with interfacetal setae. Antennal insertion located anterolaterally, well separated, dorsally concealed by weak protuberances. Antenna 11‐segmented, with loose 2‐segmented club (somewhat vaguely defined, as the antennomere 7 or 9 is also wider than the preceding antennomere); antennomeres 1 and 2 elongate, relatively stout; antennomeres 3–5 elongate, shorter and narrower than 2; antennomeres 6–9 submoniliform; antennomeres 7 and 8 wider than 6; antennomere 9 wider than 7 and 8; antennomeres 10 and 11 wider than all previous antennomeres; antennomere 11 as wide as and longer than 10 and with short but distinct basal stalk. Labrum transverse, with anterior edge multidentate. Mandibles falciform, long and slender, appearing generally symmetrical (but asymmetrical in details); inner edge with one prominent tooth and otherwise smooth. Right mandible lower than left mandible when mandibles closed, with dorso‐lateral groove to receive left mandible. Maxillary palpi 4‐segmented; palpomere 3 longer than 2, distinctly fusiform; palpomere 4 aciculate. Labium anteriorly with deep median notch; labial palpi 3‐segmented, inserted widely apart (nearer sides than middle of labium); palpomere 2 longer and distinctly wider than 1; palpomere 3 aciculate. Mentum trapezoidal, 2.5 times as wide as long, with entire surface in same plane (palpomere rests absent). Submentum without transverse ridge and without any cuticular projections. Gular sutures fused posteriorly, abruptly diverging anteriorly. Neck region ventrally with distinctive squamous sculpture.

Pronotum about 1.05 times as wide as long, sides weakly convex, abruptly constricted posteriorly; disc smooth, without grooves or clear punctation; lateral pronotal carinae present, complete. Prosternum unmodified, smooth, without medial carinae or other special structures; anterior margin evenly smooth (not dentate); basal margin with well‐developed anteprocoxal lobes and arcuate anteprocoxal carina. Procoxae small, narrowly separated by prosternal process; procoxal fissure closed and protrochantin concealed.

Scutellar shield with exposed portion subtriangular. Elytra longer than pronotum, posteriorly truncate, apicolaterally with elytro‐tergal locking notch, with sides broadly curving posteriorly; surface smooth. Hind wings well developed. Mesoventrite with only weakly defined posteromedial longitudinal bump, without special carinae or other structures; mesoventral process overlapping anterior process of metaventrite ventrally and narrowly separating mesocoae. Metaventrite without carinae or other special structures. Stem of metendosternite present. Metacoxae slightly wider than long, laterally relatively abruptly narrowed, not reaching elytra laterally, very narrowly separated.

Legs slender. Tibiae with stout setae near apex. Tarsi 4‐4‐4 (due to imperfect fusion of original tarsomeres 1 and 2, as judged by positions of seemingly serially homologous setae); all tarsomeres simple; last tarsomere longer than penultimate and antepenultimate tarsomeres combined. Pretarsal claws simple, without visible empodial setae.

Abdomen with two pairs of subparallel‐sided parasclerites at least on segments IV–VI; inner parasclerite wider than outer parasclerite. Intersegmental membranes attached apically to preceding segment, with minute hexagonal sclerites in “brick‐wall” pattern. Spiracles of segments III–IV located near middle of sides of tergites. Sternite III with sharp intercoxal carina. Sternite VIII with apex produced. Second gonocoxites acutely pointed, without gonostyli.

## 4 Discussion

*Zavoticus* gen. nov. clearly belongs to the subfamily Euaesthetinae due to its denticulate labral margin, acutely pointed second gonocoxites without gonostyli, well‐developed pronotosternal suture, closed procoxal fissure with concealed trochanters, slender curved mandibles, and clubbed antennae (Clarke and Grebennikov 2009). *Zavoticus* can be separated from all other genera of Euaesthetinae based on the comparison of the following characters.

Genera of Nordenskioldiini, Stictocraniini, and Stenaesthetini, as well as *Alzadaesthetus chilensis* Kistner, have 5‐segmented tarsi on the fore and mid legs (Scheerpeltz 1974; Newton et al. 2000; Clarke 2018). In contrast, similar to the remaining euaesthetines, the tarsomeres 1 and 2 appear to be fused in all legs of *Zavoticus*, although the edges between the original tarsomeres 1 and 2 are still somewhat distinct, especially as seen in the mesotarsus. Described members of Stictocraniini, Stenaesthetini, Austroesthetini (except for *Nothoesthetus* Sáiz) and *Alzadaesthetus furcillatus* Sáiz have no parasclerites on abdominal segments IV–VI (Clarke and Grebennikov 2009), while in *Zavoticus* the tergum and sternum of these segments are divided by parasclerites. *Zavoticus* additionally differs from Stenaesthetini and *Fenderia* Hatch by the absence of the dorsolateral carina of the head, and from various euaesthetine genera including *Alzadaesthetus* Kistner, *Tasmanosthetus* Puthz, and *Nothoesthetus* by the well‐developed hind wings.

Among the currently recognized tribes, *Zavoticus* appears to fit best in the Euaesthetini, although the tribe as currently circumscribed is likely to be non‐monophyletic (Clarke and Grebennikov 2009). *Zavoticus* has no transverse carina on the submentum. Among the euaesthetine genera sampled in the phylogenetic analyses, the absence of submental transverse carina is otherwise known only in *Alzadaesthetus furcillatus* and Euaesthetini (Clarke and Grebennikov 2009). Within Euaesthetini, limited information is available for the rare genera *Ctenomastax* Kraatz, *Euaesthetotyphlus* Coiffait & Decou, *Macroturellus* Orousset, *Schatzmayrina* Koch, and *Tamotus* Schaufuss. Nevertheless, *Schatzmayrina* differs from *Zavoticus* by the 9‐segmented antennae with 1‐segmented club and labrum with nearly smooth anterior edge (Coiffait 1984; Clarke and Grebennikov 2009). *Tamotus* differs from *Zavoticus* by the weakly sclerotized and strongly bilobed penultimate tarsomeres (Puthz 1973). *Ctenomastax* differs from *Zavoticus* by the larger and sparser teeth along the anterior edge of the labrum (Scheerpeltz 1974; Puthz 1988: figure 2). *Euaesthetotyphlus* differs from *Zavoticus* by the reduced eyes and wings (Coiffait and Decou 1970; Coiffait 1984). *Macroturellus* differs from *Zavoticus* by the smooth labral margin and multiple structural characters shared with many *Octavius* species (Orousset 1987).

**FIGURE 5:**
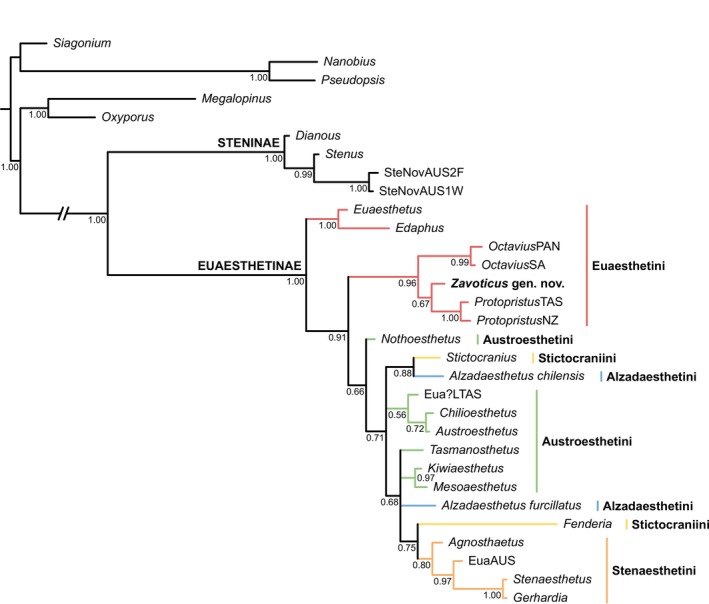
Suggested placement of *Zavoticus* gen. nov. within Euaesthetinae. Tree resulting from constrained Bayesian analysis.

**FIGURE 6:**
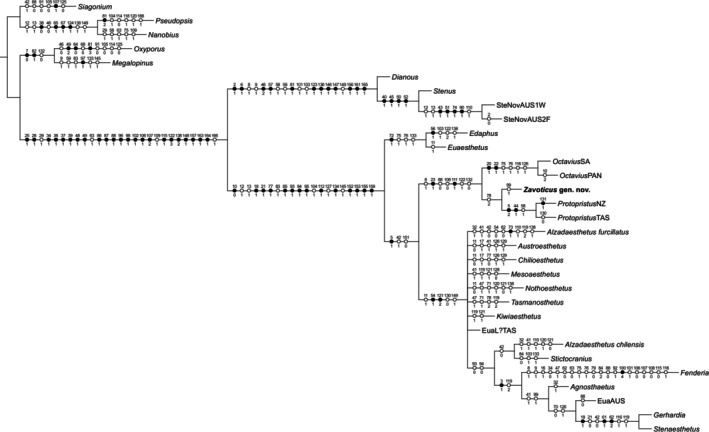
Suggested placement of *Zavoticus* gen. nov. within Euaesthetinae. Tree resulting from constrained parsimony analysis under implied weighting.

*Zavoticus* shares with *Octavius* and *Protopristus* Broun three characters that are not or rarely known in other Euaesthetini and Euaesthetinae: (1) the presence of a distinct metendosternal stalk, which is absent (or very short) in the majority of Euaesthetinae, including *Edaphus* Motschulsky and *Euaesthetus* Gravenhorst (Clarke and Grebennikov 2009; e.g., Orousset 1988: figure 299; Byeon et al. 2022: figure 2); (2) the presence of two pairs of parasclerites on abdominal segments IV–VI, whereas most Euaesthetinae, including *Edaphus* and *Euaesthetus*, have only one pair or none; and (3) the apical attachment of abdominal intersegmental membranes to the preceding segment, while in the majority of Euaesthetinae, including *Edaphus* and *Euaesthetus*, the abdominal intersegmental membranes attach subapically to the preceding segment and are thus partially concealed (Clarke and Grebennikov 2009). *Zavoticus* further agrees with these two genera in general habitus (long, slender and parallel‐sided) and head with a distinct lateral constriction of neck and temples behind the eyes. Thus, we feel confident that *Zavoticus* fits comfortably alongside these two genera in what we will refer to as the *Octavius* generic group, as supported by our phylogenetic analyses (Figures 5 and 6).

According to the key and character matrix by Clarke and Grebennikov (2009), *Zavoticus* differs from *Protopristus* by the largely united gular sutures (united anteriorly only in *Protopristus*; completely separate in *Euaesthetus* and *Edaphus*), and differs from *Octavius* by the absence of oblique carinae and lateral transverse carinae on mesoventrite (present in *Octavius*, *Edaphus* and *Euaesthetus*) and distinctly separated antennomeres 10 and 11 (immovably fused in nearly all *Octavius*). However, a clear separation from *Octavius* is more problematic than it seems, mainly due to the broad modern taxonomic concept of extant members of this genus introduced by Puthz (1977, 1980) and followed and extended by others (e.g., Orousset 1988, 2012) who have extensively discussed morphological variations within the genus in shape, surface sculpture, and many systematic characters formerly used to define genera. In this modern concept, *Octavius* is a nearly worldwide genus of 262 extant and one extinct named species (Newton 2022). Its diverse morphology is exemplified by the fact that these species were originally described in eight different genera, now considered synonyms (e.g., Puthz 1980; Orousset 1988). For example, as noted by Clarke and Chatzimanolis (2009), some modern European species such as *O. confusus* Coiffait and *O. grandiceps* (Mulsant & Rey) and the extinct species *O. electrospinosus* from Kachin amber have antennomeres 10 and 11 distinctly separated, similar to our fossil species. Likewise, the mesoventral carinae are difficult to see even on modern slide‐mounted specimens and are not present in some extant species of *Octavius* examined by one of us (A.F.N.) on slides.

Nevertheless, *Zavoticus* can be confidently separated from *Octavius* (and also *Protopristus*) by having only a very weak and indistinct dorsal nuchal groove or depression (Clarke and Grebennikov 2009), and possibly by the complete second more lateral pair of paratergites (this second pair is present only as minute triangular slerites in the apical half of the segment in six extant species examined by us on slides). All the specimens of extant *Octavius* (including more than 50 species) checked by us (A.F.N./D.J.C.) have a distinct nuchal groove separating head and neck dorsally. The fossil *O. electrospinosus* also has a distinct nuchal groove, according to the original description and drawing (Clarke and Chatzimanolis 2009), and as recently reverified by one of us (D.J.C.) on the type specimen.

In agreement with the above discussion of morphological characters, our phylogenetic analysis (Figures 5 and 6) suggests that *Zavoticus* is sister to *Protopristus* (albeit only weakly supported) and clearly belongs to the *Octavius* generic group (strongly supported). Certainly, the generic concepts within this group, and the relationship of this group to other Euaesthetini and Euaesthetinae, need much further study. Thus, this result should be viewed as preliminary only. Most of the euaesthetine tribes are non‐monophyletic in morphology‐based analyses (as in Clarke and Grebennikov 2009), and the relationships among the euaesthetines have not yet been extensively investigated based on molecular data. A robust molecular phylogenetic framework of Euaesthetinae will be helpful to further determine the systematic position of *Zavoticus*.

## Author Contributions

**Yan‐Da Li:** conceptualization (equal), data curation (equal), formal analysis (equal), investigation (lead), visualization (equal), writing – original draft (equal), writing – review and editing (equal). **Dave J. Clarke:** formal analysis (equal), investigation (lead), writing – review and editing (equal). **Alfred F. Newton:** investigation (lead), writing – review and editing (equal). **Di‐Ying Huang:** funding acquisition (equal), investigation (equal), writing – review and editing (equal). **Chen‐Yang Cai:** conceptualization (equal), funding acquisition (equal), investigation (equal), supervision (equal), writing – review and editing (equal).

## Conflicts of Interest

The authors declare no conflicts of interest.

## Supporting information


**File S1:** Morphological data matrix.
**File S2:** R script for constrained parsimony analysis.

## Data Availability

The data matrix for the phylogenetic analyses is available in the Supporting Information. The original confocal data are available in the Zenodo repository (https://doi.org/10.5281/zenodo.16218950).
